# Unspoiled Identities of People Living Alone With Dementia: Resisting Stigma by Helping Others

**DOI:** 10.1093/geroni/igab046.1387

**Published:** 2021-12-17

**Authors:** Kate de Medeiros, Laura Girling

**Affiliations:** 1 Miami University, Oxford, Ohio, United States; 2 University of Maryland, Baltimore County, Baltimore, Maryland, United States

## Abstract

Goffman (1963) described stigma as the shift from being viewed as a whole and usual person to one with a spoiled identity. People living with dementia (PLWD) often report feeling stigmatized. Many dementia stereotypes highlight losses (e.g., loss of self) and negatively position the person as a passive, dependent care recipient. Here, we present findings from a qualitative study of people living alone with dementia (N=10) in the community that challenge these stereotypes. Analysis of in-depth interviews revealed that many participants resisted the spoiled identity label through active engagement in the community such as participating in paid employment, providing care for neighbors and family members, and volunteering. Overall, findings underscore the need to rethink and challenge common perceptions of PLWD that are focused solely on care, to recognize their active and valuable role in the lives of others. How PLWD negotiate these identities should inform policies of dementia in community.

